# Abnormalities in the Visual Processing of Viewing Complex Visual Stimuli Amongst Individuals With Body Image Concern 

**DOI:** 10.5709/acp-0185-0

**Published:** 2016-03-31

**Authors:** A. J. F. Duncum, K. J. Atkins, F. L. Beilharz, M. E. Mundy

**Affiliations:** School of Psychological Sciences and Monash Institute of Cognitive and Clinical Neurosciences, Monash University

**Keywords:** body image, visual processing, inversion effect, perceptual learning, faces, bodies, scenes, objects, body dysmorphic disorder

## Abstract

Individuals with body dysmorphic disorder (BDD) and clinically concerning body-image concern (BIC) appear to possess abnormalities in the way they perceive visual information in the form of a bias towards local visual processing. As inversion interrupts normal global processing, forcing individuals to process locally, an upright-inverted stimulus discrimination task was used to investigate this phenomenon. We examined whether individuals with nonclinical, yet high levels of BIC would show signs of this bias, in the form of reduced inversion effects (i.e., increased local processing). Furthermore, we assessed whether this bias appeared for general visual stimuli or specifically for appearance-related stimuli, such as faces and bodies. Participants with high-BIC (*n* = 25) and low-BIC (*n* = 30) performed a stimulus discrimination task with upright and inverted faces, scenes, objects, and bodies. Unexpectedly, the high-BIC group showed an increased inversion effect compared to the low-BIC group, indicating perceptual abnormalities may not be present as local processing biases, as originally thought. There was no significant difference in performance across stimulus types, signifying that any visual processing abnormalities may be general rather than appearance-based. This has important implications for whether visual processing abnormalities are predisposing factors for BDD or develop throughout the disorder.

## Introduction

Body image is a multi-dimensional construct that can be defined as the elastic and changeable beliefs and subjective emotions surrounding the degree of satisfaction an individual has with their appearance (Cash, Phillips, Santos, & Hrabosky, 2004). Body image exists on a spectrum of body image concern (BIC; Callaghan, Lopez, Wong, Northcross, & Anderson, 2011), which is defined as the level of concern an individual has regarding the appearance of her/his own body, and ranges from healthy to unhealthy (Mundy & Sadusky, 2014). High BIC is an unhealthy, distorted state, distinguished by high levels of dysmorphic concern (Akbarbegloo, Habibpur, & Motaarefi, 2010). Dysmorphic concern is a preoccupation or intense concern with a perceived defect in appearance (Littleton, Axsom, & Pury, 2005).

Along with the numerous psychosocial impairments, high BIC has been linked to various psychological disorders, including body dysmorphic disorder (BDD; Rosen & Ramirez, 1998). At the core of BDD is a fundamental disturbance of the emotional, behavioural, and cognitive components of body image (Hrabosky et al., 2009). BDD is characterised by a preoccupation with defects in one’s appearance that are, to others, minor or non-existent (American Psychiatric Association, 2013). With this fixation on perceived body flaws comes significant distress, disability, and functional impairment. Repetitive and compulsive behaviours are performed, including mirror checking, camouflaging, and excessive grooming (Crerand & Sarwer, 2010). Prevalence is estimated to range from 1.8% (Buhlmann et al., 2010) to 2.3% (Bartsch, 2007), with approximately equal numbers of males and females (Koran, Abujaoude, Large, & Serpe, 2008). Individuals with BDD are more likely to be house-bound and hospitalised, and have a greater risk of suicidal ideation, suicidal attempts, and comorbid mental disorders (Buhlmann et al., 2010; Gustad & Philips, 2003; Phillips et al., 2006).

Recent studies have suggested that patients with BDD may also show abnormal visual processing mechanisms (Feusner, Bystritsky, Hellemann, & Bookheimer, 2010; Feusner, Hembacher, Moller, & Moody, 2011; Feusner, Moller, et al., 2010). A bias towards local, detail-based processing for certain stimuli has been demonstrated in BDD patients, compared to the normal predisposition toward configural, global processing in healthy people. This bias, along with a maladaptive overall body image, may relate to the pathological fixation on minor details of appearance, influencing the belief that these areas are flawed (Mundy & Sadusky, 2014).

Global processing involves perceiving the “whole” stimulus; it is a top-down, automatic process, characterised by perceiving associations among stimulus features (Minnebusch & Daum, 2009). It is the more commonly utilised mechanism for stimulus recognition as it results in faster image processing and is therefore of perceptual advantage (Proverbio, Minniti, & Zani, 1998). Conversely, local processing refers to the perception of stimulus details, as it is a bottom-up method that involves feature-based processing mechanisms (Minnebusch & Daum, 2009). Once individuals commence visual processing, whichever mechanism is best suited to the demands of the stimulus is then consequently employed (Bouvet, Rousset, Valdois, & Donnadieu, 2011).

The nature of global and local processing can be examined via stimulus inversion effects, in particular the face-inversion task (Yin, 1969). The face-inversion effect is a robust phenomenon where faces are disproportionately difficult to recognise while inverted as opposed to upright (Farah, Tanaka, & Drain, 1995). Inverted faces are subject to less accurate and slower visual processing compared to their upright counterparts (Reed, Stone, Bozova, & Tanaka, 2003), which is believed to be due to a disruption of global processing mechanisms (Farah et al., 1995; Freire, Lee, & Symons, 2000). Global processing relies on a representation of the overall stimulus, which is formed via the configural relationships between features of the stimulus (e.g., the distance between and relative spatial location of the eyes, nose, and mouth). When a face image is inverted this typical structural arrangement is disrupted, forcing individuals to rely on perceiving the individual features and piecing these together—that is, engaging in local processing. Overall, this results in longer processing times and less accurate recognition of inverted faces (Reed, Stone, Grubb, & McGoldrick, 2006), as initial fast global, configural, processing must be replaced by slower, piecemeal local processing. Inversion effects have also been shown in other complex visual stimuli such as bodies, objects, and scenes (Bruce, Doyle, Dench, & Burton, 1991; Epstein, Higgins, Parker, Aguirre, & Cooperman, 2006; Reed et al., 2003, 2006), albeit with weaker effects than found in faces.

Neuroimaging studies have found evidence for the inversion effect, with different brain regions activated during the processing of inverted and upright faces and scenes, demonstrating a disruption of regular global processing when images are inverted (Epstein et al., 2006; Haxby et al., 1999). The lateral occipital cortex (LOC; Malach et al., 1995), thought to be selective to non-face objects, was activated in response to inverted faces in addition to the fusiform face area (FFA; site of face perception; Haxby et al., 1999; Kanwisher, McDermott, & Chun, 1997). Neurophysiological studies also demonstrated this abnormality; inverted faces elicit a delayed but enhanced N170 event-related peak compared to upright faces, with the N170 localised to the LOC and FFA areas (Herrmann, Ehlis, Muehlberger, & Fallgatter, 2005; Itier & Taylor, 2004). The N170 is thought to be produced by the generation of global face configurations at the later stages of structural encoding (Jacques & Rossion, 2007), while a delayed peak is elicited by images lacking these global features (Eimer, 2000). Neurophysiological studies have also demonstrated similar irregularities in the N170 peak for body and object stimuli (Rossion, Joyce, Cottrell, & Tarr, 2003; Tao, Zeng, & Sun, 2014).

Additionally, scene inversion alters recognition and elicits gaze scan-paths similar to those expected when processing local information (Harding & Bloj, 2010). Compared to upright scenes, inverted scenes involve increased neural activation in the LOC while activity in the parahippocampal place area (PPA; processes structure of scenes and places; Epstein & Kanwisher, 1998) decreases, indicating a disruption of regular neural activity (Epstein et al., 2006). It may be concluded from these neurological studies that activity in the LOC is indicative of inverted images being processed more as generic objects, rather than as specific stimulus types for which there may be a global template. If a local processing bias does indeed exist when viewing face images in individuals with BDD (Feusner et al., 2011; Feusner, Moller, et al., 2010), as the evidence above suggests, it logically follows that the same bias may occur for other stimulus types.

Individuals with BDD are believed to visually process via a local bias leading to decreased susceptibility to the stimulus inversion effect. This bias has been demonstrated by Feusner, Moller, et al. (2010) who found that healthy controls were more adversely affected by the face inversion effect than clinical BDD participants. Subjects were shown a single face image followed immediately by two, either upright or inverted, identical or dissimilar faces, and asked to select the identical face as quickly and accurately as possible. Although, there were no differences between the groups for upright face recognition, healthy controls performed far worse on inverted face trials, indicating that the inversion effect was significantly reduced in individuals with BDD. Given this significant deviation from the norm, the authors proposed a local processing bias in the BDD group: that they process face stimuli predominantly with a bottom-up bias, regardless of orientation. Healthy controls experienced a longer time delay and greater inaccuracy as they switched from their default global processing of upright faces to local processing of the inverted faces, while those with BDD lacked these measurement disparities due to their consistent local bias. Further supporting the local bias hypothesis, individuals with BDD are also able to recognise inverted famous faces with greater accuracy than healthy controls when presented for an unlimited time (Jefferies, Laws, & Fineberg, 2012), and additionally have a heightened sensitivity to subtle changes in others’ facial features (Stangier, Adam-Schwebe, Müller, & Wolter, 2008).

This psychophysical research is further supported by various neuroimaging studies which have found hypoactivity and hyperactivity in various stages of the visual system, including a general left hemisphere dominance (local processing) for visual perception, (Feusner et al., 2011; Feusner, Townsend, Bystritsky, & Bookheimer, 2007). Feusner et al. (2011) have suggested that the reduction in neural activity in the secondary stages of visual processing may be related to a reduction in global processing, and that heightened activity in other stages may be associated with an increase in local processing. Functional magnetic resonance imaging (fMRI) studies have also indicated that individuals with BDD may visually process faces using local processing neural mechanisms, regardless of whether there is local or global information present (Feusner, Moody, et al., 2010; Feusner et al., 2007). It should also be noted that children show a reduced face inversion effect and dominant detail-based processing (Aylward et al., 2005; Joseph et al., 2006), similar to the BDD patients. As such, these abnormalities may be the result of abnormal neural development that could potentially lead to the presence of a predisposing local bias.

Abnormal visual processing has also been noted for non-face stimuli. Individuals with BDD displayed hypoactivity in global processing areas when viewing houses within a scene (Feusner et al., 2011). Activity in areas associated with extracting global information from objects was also shown to decrease with symptom severity. Participants with BDD have been found to focus more on isolated details rather than the global organisational features of the Rey-Osterrieth figure copy task (Deckersbach et al., 2000). The presence of a local bias for complex objects as well as BDD symptom-related stimuli (such as faces) can be taken as evidence for a general visual processing bias. Though the presence of a local bias in other stimulus types (e.g., bodies, scenes) has yet to be fully investigated, an overall brain network organisation, in which local connections dominate, has been demonstrated in BDD (Arienzo et al., 2013). This neural configuration would provide an environment for an imbalance between local and global visual processing which, logically, would not be specific to stimulus type.

An overall flaw in the research investigating visual processing in individuals with BDD (often acknowledged by the authors), is the recruitment of patients with severe BDD as this limits the interpretation and generalisation of the results (Feusner, Moller, et al., 2010). Whether these visual processing discrepancies exist for those with milder BDD, or in others on the BIC spectrum, that do not have clinical levels of dysmorphic concern, has received little attention. It is important to understand the nature of a local perceptual bias, and whether it may exist in the wider population as a marker of BIC. Furthermore, it is also unclear whether these visual abnormalities precede and contribute to the development of BDD (i.e., the bias towards local processing contributes to the intense appraisal of one’s own appearance) or are a result of the disorder (i.e., the fixation on perceived defects contributes to a bias of detail-focussed visual processing). The generalisability of this perception bias to a variety of stimuli (both appearance and non-appearance related) has also very rarely been examined. In the general population, dysmorphic concern is not associated with attention to appearance related features when visual stimuli are presented for short durations (Onden-Lim, Wu, & Grisham, 2012). As such, any biases that exist for non-clinical populations may be general, rather than only for appearance-related images. The existence of a general, as opposed to a body- or face-specific visual bias, may provide initial evidence that such a bias pre-exists specific BIC and may contribute to its development.

The present study drew from the protocol of Mundy and Sadusky (2014), but sought to address the limitations of previous research by recruiting a non-clinical sample, whilst excluding those that might have clinically concerning but undiagnosed levels of BIC. This strategy sought to discover whether or not a visual processing bias exists in an otherwise healthy population (and thus perhaps contributes to progression of BDD symptoms). Individuals with high and low BIC viewed images of faces, bodies, objects, and scenes to explore any processing defects for a range of stimulus types. It was hypothesised that the high BIC individuals would visually process all stimuli differently to the low BIC individuals, as shown by reduced inversion effects. More specifically, those with high BIC would demonstrate a local processing bias (faster reaction time [RT] and greater accuracy) relative to the low BIC group, when discriminating between inverted stimuli (faces, bodies, objects, and scenes).

## Experiment

### Method

#### Participants

Participants were recruited from Monash University, Clayton Campus, Australia. Interested participants were invited to complete an online questionnaire regarding their body image. A total of 371 questionnaires were submitted. Participant demographics were recorded alongside an electronic version of the Dysmorphic Concern Questionnaire (DCQ; Oosthuizen, Lambert, & Castle, 1998; described below). Participants were required to have either normal or corrected-to-normal vision; however, their visual acuity was not specifically assessed.

Forty-three participants with low DCQ scores (1-4; 13 declined to participate) and 54 participants with high DCQ scores (11-17; 29 declined to participate) were invited to participate in the behavioural task. The final sample consisted of 30 (8 males and 22 females) individuals (*M*_age_ = 22.83, *SD*_age_ = 3.86) in the low BIC group (*M*_BIC_ = 2.40, *SD*_BIC_ = 1.28), and 25 (2 males and 23 females) students (*M*_age_ = 23.08, *SD*_age_ = 5.37) in the high BIC group (*M*_BIC_ = 13.44, *SD*_BIC_ = 2.35). No individuals in the final sample reported a clinical diagnosis of BDD, though three participants had received an eating disorder diagnosis. There were no significant differences between the two BIC groups in age, *t*(53) = 0.20, two-tailed, *p* = .84. Participants who completed the behavioural portion of the experiment received 10$ as a reimbursement for their time.

#### Materials

The online questionnaire was hosted by the website Qualtrics©. The questionnaire began by asking participants if they had either normal or corrected-to-normal vision. Those that did not were directed to the end of the questionnaire. The second portion requested participants’ sex, age, and contact information, followed by the DCQ (Oosthuizen et al., 1998).

The DCQ is a seven item, four-point Likert-like scale that assesses dysmorphic concern. Participants are required to rate the extent of BIC relative to others, and past attempts to deal with any perceived physical defects. Participants rated these items from 0—*Not at all* to 3—*Much more than most people*. The item ratings were summed with possible total scores ranging from 0 to 21 (indicating low and high BIC, respectively). The DCQ was not designed as a diagnostic measure but has been used to measure clinically concerning levels of BIC, with the intention of identifying individuals requiring further assessment (Jorgensen, Castle, Roberts, & Groth-Marnat, 2001). There has been some disagreement in the literature as to the exact cut-off score for clinical concern in this measure (Mancuso, Knoesen, & Castle, 2010; Stangier, Janich, Adam-Schwebe, Berger, & Wolter, 2003). As such, the high BIC group had a restricted range of 11-17 so as to include those with high dysmorphic concern but not those at levels of most significant clinical concern. However, whilst anyone with a current or past diagnosis of BDD was excluded from the study, this range may still contain participants who could present with clinically concerning BDD. The DCQ is considered a valid (Cronbach’s α of 0.80) and reliable instrument (Jorgensen et al., 2001), making it an effective measure for the current study.

#### Stimuli

The behavioural portion of the experiment involved the presentation of a variety of stimuli: faces, bodies, objects, and scenes.

The face stimuli were created from pairs of coloured photographs of faces obtained from the Psychological Collection of Images at Stirling database (available at pics.stir.ac.uk) and the Centre for Vital Longevity Face Database (compiled for Minear & Park, 2004, available at agingmind.utdallas.edu/facedb). An equal number of male and female faces of various ethnicities with emotionally neutral expressions were used. Each original pair contained two faces with roughly similar facial features. These pairs were then morphed to increase discrimination difficulty, employing the Morpheus Photo Morpher (Version 3.16, 2011) software. For each stimulus, in each pair, a morphed image seven steps away on the morph continuum of 1-30 was chosen. This process resulted in each original stimulus pair producing two new, harder to distinguish, morphed images. The protocol of Mundy, Honey, and Dwyer (2007) was followed for the morphing process and definition of morph difficulty level. Images were cropped to remove extraneous details (e.g., hair, jewellery) and resized to ensure equal image size (325 × 500 pixels). Eight final stimulus pairs were chosen containing an original and a non-matching, morphed, face.

The body stimuli were created using the software HumanCAD (Version 2.5, 2013), a human modelling program that allowed body images to be created with different proportions and postures. An equal number of male and female computerised and fully-clothed body pairs were created. These images, following the same protocol as the face stimuli, were morphed together using the Morpheus Photo Morpher (Version 3.16, 2011) software. Morphed body image pairs were chosen based on the same apparent difficulty level as the previously morphed face pairs. Images were cropped to remove the head and resized to ensure equal image size (200 × 500 pixels). Eight final stimulus pairs were chosen, containing an original image and its morphed partner.

The scene stimuli (797 × 448 pixels) were eight pairs of coloured, realistic computer-generated, outdoor scene images (e.g., mountain ranges) that were created using Vue Pioneer (Version 9, 2010), a 3D modelling software. An original image was created and then manipulated using the software (e.g., shifting a coast line) to create a partner image with high discrimination difficulty (for a detailed description of this procedure see Mundy, Downing, Dwyer, Honey, & Graham, 2013). All the pairs were non- matching, containing one original image and its manipulated partner image.

The object stimuli (300 × 300 pixels) were eight pairs of black and white, computer-generated, nonsense images with a strict prototypical structure (including base, central mass, and appendage), which implied a correct orientation, created by combining multiple 3D objects using Adobe Photoshop. Nonsense objects were chosen rather than generic items, as familiarity with stimuli has been shown to affect inversion, and this may differ between participants (Diamond & Carey, 1986). Following the same procedure as the scene stimuli, initial images were created and manipulated to create a partner image with high discrimination difficulty. The pairs all contained non-matching images; one initial image and its manipulated partner image.

**Figure 1.**
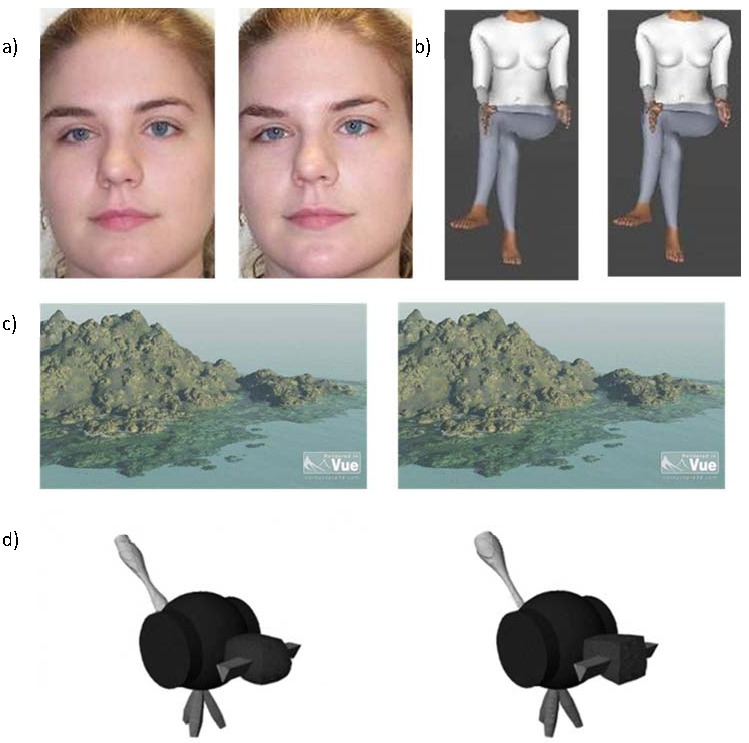
Examples of difficult to discriminate (non-matching), face (a), body (b), scene (c), and object (d) stimulus pairs used in the behavioural task.

Similar to previous studies in this area (e.g., Mundy & Sadusky, 2014), the on screen dimension for all images was 15 × 12 degrees of visual angle (height × width). Pilot testing (*n* = 2) was used to ensure stimuli had a similar level of discrimination difficulty. An example of difficult to discriminate (non-matching) stimulus pairs for each stimulus type can be seen in Figure 1. The stimuli were presented and RTs and accuracy were recorded using Presentation (Version 14.9, 2010) on a compatible PC.

#### Procedure

Participants completed the online questionnaire and their DCQ score was calculated. Those with a score in the required range were invited into the laboratory to complete the behavioural portion of the experiment, which was an upright-inverted discrimination task. Participants were seated 70 cm away from a 24 inch PC monitor in a darkened room. Participants were informed that they would see successive stimuli appear on the screen and that they must decide as quickly and as accurately as possible if the stimulus pairs were the same or different (right and left mouse buttons, respectively). They were warned that any differences within the stimulus pairs would be subtle.

Each trial began with the presentation of the first stimulus for 500 ms, followed by a blank screen for 300 ms, and then the second stimulus for 500 ms. An inter-trial interval (blank screen) of 3-6 s separated each trial. The participants’ response period began 300 ms into the second stimulus presentation, in order to discount any anticipation errors. The response period ended after 3 s and the following trial started regardless of whether participants selected a response or not. Trials were discarded if no response was given.

**Figure 2.**
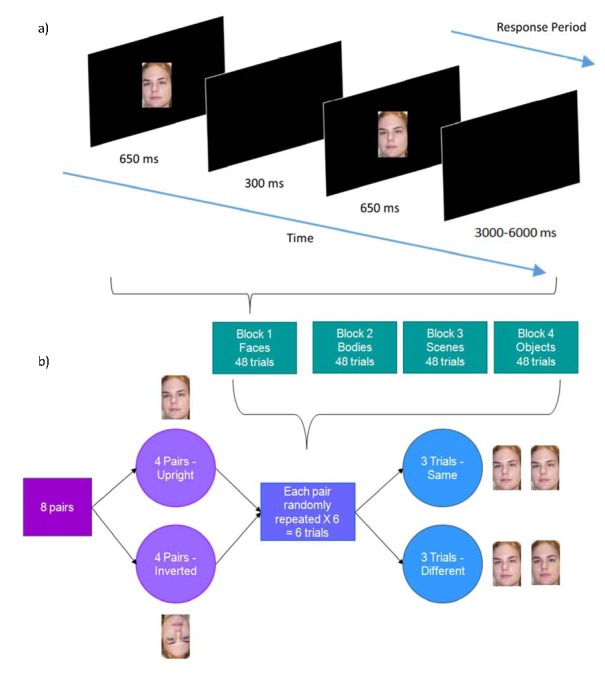
Schematic diagram of stimulus presentation for a single trial in the upright-inverted discrimination task (a). The response period lasted for 3,350 ms. Schematic diagram of conditions and trials in the face-stimulus block of the behavioural task (b). Conditions in the other stimulus blocks were created in an analogous fashion (not depicted).

The stimulus sets (faces, bodies, scenes, and objects) were shown in four blocks of trials containing only one type of stimulus. These blocks were presented in a counterbalanced order across participants. Each block contained eight unique stimulus pairs. Four were presented consistently upright and four consistently inverted at 180º. Each pair was presented six times. In three of these trials the pair contained non-matching stimuli (the original stimulus and its morphed partner or vice versa) and required participants to respond “different”. The remaining three trials presented matching stimuli (the original stimulus and its exact copy, or the morphed stimulus and its exact copy) and required the participants to respond “same”. This paradigm resulted in 48 randomized trials per block, for a total of 192 trials. A diagram of the overall stimulus presentation can be seen in Figure 2. The behavioural task of this experiment took approximately 35 min.

### Results

For each participant, mean discrimination accuracy and RT were recorded for each condition. Accuracy was calculated as a percentage of correct responses out of the total number of trials for each stimulus block. RT was only analysed for the correct responses. SPSS statistics version 19 (IBM, Australia) for Windows was used to run all statistical analyses. An alpha level of .05 (two-tailed) was set for all of the analyses, unless noted otherwise. The three high BIC participants who had previously been diagnosed with an eating disorder were removed from the analysis to ensure effects relating to eating disorders did not affect the outcome of the primary focus on BIC. Four outliers with extreme low scores were identified with z-scores below -3.29, and they were dealt with via the winsorising method (Tabachnick & Fidell, 2014).

#### Accuracy Analysis

**Figure 3.**
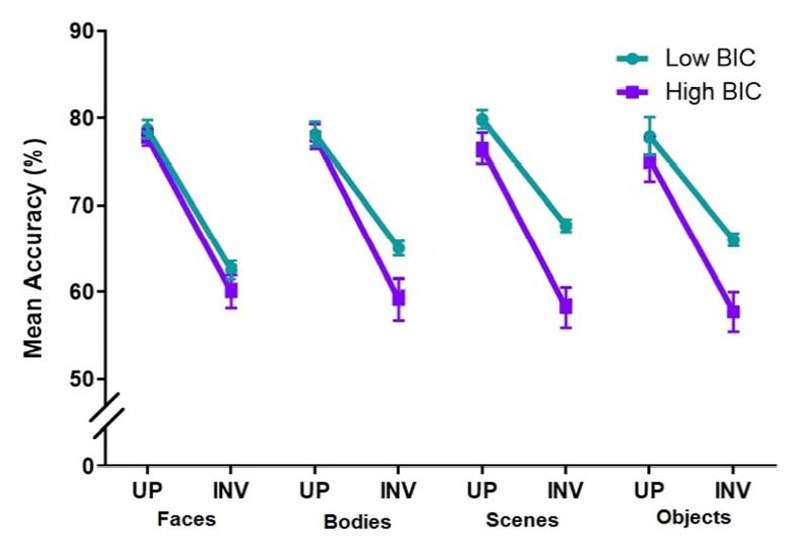
Percent discrimination accuracy for individuals with low and high body image concern (BIC) during upright (UP) and inverted (INV) presentation of various stimuli. Error bars represent ±1 SEM. Points are horizontal so that error bars are visible.

A mixed-measures analysis of variance (ANOVA), with the repeated-measures variables Stimulus (faces, bodies, scenes, objects) and Orientation (upright, inverted) and the between-groups variable BIC (low, high) was performed on the accuracy data. The assumption of sphericity was satisfactorily met, however, normality, homogeneity of covariance, and homogeneity of variance were not. Due to the robustness of the factorial ANOVA performed, and the moderate sample size, these violations were considered acceptable (Field, 2013; Hills, 2011; Tabachnick & Fidell, 2014). The interaction between Stimulus, Orientation, and BIC level was not significant, *F*(3, 150) < 1, *p* = .43 (*ns*). This data is summarised in Figure 3.

There was a significant main effect for Orientation, *F*(1, 50) = 280.94, *p* < .001, η_p_^2^ = .85. This indicates that there was an overall standard inversion effect; upright stimuli had greater discrimination accuracy when compared to inverted stimuli, *M*_diff_ = 15.93, *SE* = 0.95, *p* < .001, 95% CI [14.02, 17.84]. Overall the low BIC group discriminated between stimuli with greater accuracy compared to the high BIC group, *F*(1, 50) = 21.58, *p* < .001, η_p_^2^ = .30. There was also a significant interaction between Orientation and BIC, *F*(1, 50) = 7.1, *p* = .01, ηp2 = .12. There was no significant main effect of Stimulus, *F*(3, 150) < 1, *p* = .79 (*ns*), or Stimulus and BIC level interaction, *F*(3, 150) = 1.16, *p* = .33, η_p_^2^ = .02. These results indicate that performance did not vary based on stimulus type and this did not change regardless of BIC level. The interaction between Stimulus and Orientation was also not significant, *F*(3, 150) < 1, *p* = .40 (*ns*).

The significant interaction between BIC and Orientation was further explored by simple effects analysis performed separately for the upright and inverted stimuli, using independent error terms and a Bonferroni adjusted alpha of .025 to maintain the family wise error rates at .05. Each simple effect analysis was followed by simple contrasts comparing low and high BIC using an adjusted alpha of .013. The simple effect for the upright stimuli was not significant, *F*(1, 50) = 1.45, *p* = .23, η_p_^2^ = .03; with no difference seen between high and low BIC groups when viewing upright stimuli, *M*_diff_ = 1.64, *SE* = 1.36, *p* = .23, 95% CI [-1.09, 4.47]. For the inverted stimuli, further analysis revealed a significant effect *F*(1, 50) = 28.66, *p* < .01, η_p_^2^ = .36. The low BIC individuals, with significantly greater discrimination accuracy, *M*_diff_ = 6.71, *SE* = 1.25, *p* < .01, 95% CI [4.19, 9.22], were less affected by inversion compared to the high BIC individuals.

#### Reaction time Analysis

A mixed-measures ANOVA, with the repeated-measures variables Stimulus (faces, bodies, scenes, objects) and Orientation (upright, inverted), and with the between-groups variable BIC (low, high) was performed on the RT data. The assumptions of sphericity, homogeneity of covariance, and homogeneity of variance were satisfactorily met, however, the assumption of normality was not. Due to the robustness of the factorial ANOVA performed, and the moderate sample size, it was assumed this violation would have little effect (Field, 2013; Hills, 2011; Tabachnick & Fidell, 2014). The interaction between Stimulus, Orientation, and BIC level was not significant, *F*(3, 150) < 1, *p* = .99 (*ns*). This data is summarised in Figure 4.

The main effects of Stimulus, Orientation, and BIC level were not significant, *F*(3, 150) < 1, *p* = .60 (*ns*), *F*(1, 50) < 1, *p* = .72 (*ns*), *F*(1, 50) <1, *p* = .80 (*ns*), respectively. The interactions between Stimulus and BIC level, Orientation and BIC level, and Stimulus and Orientation were also not significant, *F*(3, 150) < 1, *p* = .91 (*ns*), *F*(1, 50) = 1.93, *p* = .17, η_p_^2^ = .04, *F*(3, 150) < 1, *p* = .51 (*ns*), respectively.

## Discussion

This study sought to determine whether a general local processing bias existed in a non-clinical sample of individuals with high and low levels of BIC. Significant differences were only found in the accuracy rates of participants and not the RT data. As such, conclusions drawn about the proposed relationship—that high BIC individuals would demonstrate a local processing bias for all stimulus types—will mostly be based on the accuracy data. A standard inversion effect was seen for all stimuli in both BIC groups, consistent with previous studies in healthy individuals (Reed et al., 2003; Yin, 1969). High BIC participants did not differ in their discrimination accuracy for any of the stimulus types, supporting the theory that any perceptual abnormalities may be general rather than appearance-related. The hypothesised relationship between high BIC participants and a reduced inversion effect compared to low BIC participants was not supported. Instead, the low BIC group displayed a reduced inversion effect (greater accuracy) across all stimuli when compared to the high BIC group. Through further investigation it became clear that although both groups process upright stimuli with similar levels of accuracy, our low BIC group responded more accurately to inverted stimuli than the high BIC group (contrary to expectations). As such, individuals with high BIC appear to experience a greater inversion effect, suggesting any visual bias in this group is potentially more complex than initially thought.

The comparable response rates for the various stimulus types for individuals with high BIC suggest consistent perceptual processes across the different complex stimulus types presented. As such, any visual processing abnormalities present in the high BIC group may indeed be general rather than appearance-specific. This is consistent with previous research that found no selective attention bias for appearance-related stimuli in individuals with high levels of dysmorphic concern, with a stimulus presentation similar to that of the current study (Onden-Lim et al., 2012). Neuroimaging evidence has also demonstrated similar patterns of abnormal neural activation for both appearance (Feusner, Moody, et al., 2010; Feusner et al., 2007) and non-appearance (Feusner et al., 2011) stimuli in individuals with BDD, suggesting a more universal stimulus deficit. This deficit would be present (if not always overtly visible) regardless of stimulus presentation times and as such further research is needed to examine this area.

This study’s results are, however, inconsistent with those of Mundy and Sadusky (2014) who found evidence for a local processing bias in a non-clinical population for faces, body, and scene stimuli. However, the use of a BIC group consisting of participants with highly elevated levels of dysmorphic concern and the likely presence of individuals with clinically concerning BIC (undiagnosed BDD) may partially explain the disparity between the studies. In contrast to previous research (Jefferies et al., 2012), neither the present study nor Mundy and Sadusky demonstrated any significant differences in discrimination accuracy between the low and high BIC groups for face stimuli. The lack of relationships with RT data for both high and low BIC is notable considering other studies have found differences among healthy, BDD, and high BIC participants (Mundy & Sadusky, 2014; Reed et al., 2003), sometimes at the expense of accuracy (Feusner, Moller, et al., 2010). It is unclear at this time what may be contributing to this, however, it appears in the present study participants valued accuracy over RT (perhaps due to task instructions) leading to more variable RTs.

The lack of evidence for perceptual abnormalities in scene and object stimuli in these results conflicts with BDD neuroimaging research which has demonstrated atypical neural processing of house images (Feusner et al., 2011), along with overall neural networks primed for local processing (Arienzo et al., 2013). In contrast, our results are consistent with Monzani, Krebs, Anson, Veale, and Mataix-Cols (2013) who did not find a local processing bias for non-symptom related stimuli for individuals with BDD. As with the current study, short stimulus presentation times were utilised (500 ms), which may not have allowed sufficient time, for even participants with BDD (or high BIC) to demonstrate any visual abnormalities. Participants in Feusner, Moller, et al. (2010) were also exposed to stimuli for short (500 ms) and long (5 s) durations and a local processing bias was only demonstrated for individuals with BDD during the longer presentation times. This was believed to be due to the global precedence effect, where the insufficient presentation time affords only immediate and automatic global processing techniques to occur (Bouvet et al., 2011). It is possible that in the present study, even those with a local processing bias still utilise immediate global processing initially before reverting to a more efficient local technique than their healthy counterparts. This was also seen to extend to symptom related stimuli indicating that short duration stimulus times are insensitive to the imbalance between global and local processing. Although this may seem contradictory to the results of Mundy and Sadusky (2014), who used similarly short presentation times, in that study the second stimulus appeared on the screen until participants made a response, thus the second stimulus presentation was potentially longer than 500 ms. Ultimately, it cannot be established with the current methodology at what point participants, whether high or low BIC, switch from local to global processing, or continue to process locally. Overall, the results of the present study do not provide strong support for the dominance of local processing in individuals with high BIC contrary to Mundy and Sadusky, though as an emerging research area further investigation is required before drawing any strong conclusions.

It could also be argued that the lack of support for a feature-based visual bias in this preclinical sample supports suggestions that any visual processing abnormalities seen in BDD populations are a result of the disorder, rather than contributing to its development. However, if this were the case, one would expect the high and low BIC groups to be equally susceptible to the inversion effect. This result did not occur; low BIC participants were significantly more accurate when discriminating between inverted stimuli compared to those with high BIC, and the low BIC group were more accurate for all stimuli, regardless of orientation. This difference in discrimination indicates that further research is needed to determine if the results were genuine artefacts of the experimental process and methodological limitations, or reflective of some alternate mechanism. For example, previous research employed simultaneous presentation of stimuli rather than successive presentation as in the current study. This successive presentation could have resulted in visual working memory effects which should be further examined.

An interesting potential explanation of the current findings is that rather than a local bias, individuals with high BIC (and BDD) may possess a global processing deficit. They may then be unable to fully utilise global mechanisms when viewing stimuli. As such, they may more readily and with better skill, employ slower local processing (or engage in a deliberate perceptual strategy). This notion has been previously suggested (Monzani et al., 2013) and is supported by neuroimaging studies that have demonstrated hypoactivity in global processing areas in individuals with BDD (Feusner et al., 2011; Feusner, Moody, et al., 2010). With the limited duration of stimulus presentation used here, however, individuals with high BIC may not have had enough time to employ their preferred local processing techniques effectively and were therefore reliant on their defective global perception. Thus, resulting in even lower accuracy than those with low BIC, who can still utilise (albeit attenuated in inversion) configural processing. This potential deficit in global processing may be related to a lack of insight into overall body image context that does not allow individuals with BDD to see their supposed defect in the “bigger picture” of their appearance.

Ultimately, a nonclinical sample was examined in the present study and there remains ample evidence from behavioural, psychophysiological, and neuroimaging studies to support the presence of detailed-based visual abnormalities and superior visual performance in individuals with diagnosed BDD (Deckersbach et al., 2000; Feusner, Bystritsky, et al., 2010; Feusner et al., 2011; Feusner, Moller, et al., 2010; Feusner, Moody, et al., 2010; Feusner et al., 2007). These abnormalities, however, may come about via a deficiency in global processing, which necessitates a strategy, improving or biasing the individual toward slower, more targeted local processing.

It is possible that participants in the current sample were under characterised in terms of other clinically relevant issues such as eating disorder symptomatology and issues surrounding depression and anxiety. This issue may be further compounded by research suggesting mood may alter global and local processing mechanisms (Basso, Schefft, Ris, & Dember, 1996). Future research may benefit from examining relevant clinical features alongside BIC when evaluating visual processing in this population.

Participants were also excluded from the study if they reported a BDD diagnosis and a DCQ range was chosen to exclude participants with very high levels of BIC. However, it was not within the bounds of the current study to provide clinical assessments of all participants and therefore it is possible that individuals with undiagnosed BDD were not excluded entirely. Furthermore, the present study did not assess the visual acuity of participants and as such there may be possible untested group differences.

In spite of the acknowledged limitations and unexpected results, it does appear that individuals with preclinical levels of BIC may possess patterns of visual processing abnormalities, though more research is needed to determine their exact nature. Numerous factors have been implicated in the development and maintenance of BDD, and any perceptual deficits would only be one of many contributing factors. Nonetheless, identification of a defect in the visual perception system of nonclinical individuals could lead to the earlier identification of individuals at risk of clinical conditions and inform future intervention strategies. For example, development of visual perception-related behavioural tasks or neuroimaging assessments could objectively identify those at risk of developing BDD from their performance or neural activation patterns providing a cognitive marker for the disorder. Knowledge of visual processing in these individuals could also inform treatment methods and options. By altering their visual processing, individuals with BDD may change the perception of their physical appearance.

Overall these findings suggest that individuals with high BIC process various stimuli consistently, regardless of specific type, and that any visual processing abnormalities may follow this pattern. This is an important finding, as the presence of atypical general stimulus processing suggests that this may develop pre-clinically, rather than as a result of the disorder. This would more likely be expressed as abnormalities associated with BDD symptomology. The current study’s methodology, however, has not been capable of confirming the nature of perceptual deficits in an otherwise healthy population. Ultimately, more research is needed to determine whether the perceptual abnormalities present in BDD and highly elevated dysmorphic concern populations exist in nonclinical individuals. Further examination of this will allow us to establish whether these biases contribute to the development and course of BDD or are learnt behaviours resulting from the constant attention to appearance.

## Author Note

The authors wish to express no conflict of interest and assert that all procedures contributing to this work comply with the ethical standards of the relevant national and institutional committees on human experimentation and with the Helsinki Declaration of 1975, as revised in 2008. Funding for this study was received from the School of Psychology and Psychiatry, Monash University.
